# U.S. Trends of ED Visits for Pediatric Traumatic Brain Injuries: Implications for Clinical Trials

**DOI:** 10.3390/ijerph14040414

**Published:** 2017-04-13

**Authors:** Cheng Chen, Junxin Shi, Rachel M. Stanley, Eric A. Sribnick, Jonathan I. Groner, Henry Xiang

**Affiliations:** 1 Center for Pediatric Trauma Research, The Research Institute at Nationwide Children’s Hospital, 700 Children’s Drive, Columbus, OH 43205, USA; Cheng.Chen@nationwidechildrens.org (C.C.); Junxin.Shi@nationwidechildrens.org (J.S.); Rachel.Stanley@nationwidechildrens.org (R.M.S.); Eric.Sribnick@nationwidechildrens.org (E.A.S.); Jonathan.Groner@nationwidechildrens.org (J.I.G.); 2 Center for Injury Research and Policy, The Research Institute at Nationwide Children’s Hospital, 700 Children’s Drive, Columbus, OH 43205, USA; 3 Department of Pediatrics, The Ohio State University College of Medicine, 370 West 9th Avenue, Columbus, OH 43210, USA; 4 Department of Neurosurgery, The Ohio State University College of Medicine, 370 West 9th Avenue, Columbus, OH 43210, USA; 5 Department of Surgery, The Ohio State University College of Medicine, 370 West 9th Avenue, Columbus, OH 43210, USA; 6 Division of Emergency Medicine, Nationwide Children’s Hospital, Columbus, OH 43205, USA

**Keywords:** pediatric, traumatic brain injury, emergency department

## Abstract

Our goal in this paper was to use the 2006–2013 Nationwide Emergency Department Sample (NEDS) database to describe trends of annual patient number, patient demographics and hospital characteristics of pediatric traumatic brain injuries (TBI) treated in U.S. emergency departments (EDs); and to use the same database to estimate the available sample sizes for various clinical trials of pediatric TBI cases. National estimates of patient demographics and hospital characteristics were calculated for pediatric TBI. Simulation analyses assessed the potential number of pediatric TBI cases from randomly selected hospitals for inclusion in future clinical trials under different scenarios. Between 2006 and 2013, the NEDS database estimated that of the 215,204,932 children who visited the ED, 6,089,930 (2.83%) had a TBI diagnosis. During the study period in the US EDs, pediatric TBI patients increased by 34.1%. Simulation analyses suggest that hospital EDs with annual TBI ED visits >1000, Levels I and II Trauma Centers, pediatric hospitals, and teaching hospitals will likely provide ample cases for pediatric TBI studies. However, recruiting severe pediatric TBI cases for clinical trials from a limited number of hospital EDs will be challenging due to small sample sizes. Pediatric TBI-related ED visits in the U.S. increased by over 30% from 2006 to 2013. Including unspecified head injury cases with ICD-9-CM code 959.01 would significantly change the national estimates and demographic patterns of pediatric TBI cases. Future clinical trials of children with TBI should conduct a careful feasibility assessment to estimate their sample size and study power in selected study sites.

## 1. Introduction

Traumatic brain injury (TBI) is the leading cause of death and disability in trauma patients [1]. In the United States in 2000 alone, an estimated 50,658 TBI-associated hospitalizations occurred in children ≤17 years [2]. The annual TBI-associated hospitalization rate was 70 cases per 100,000 children (≤17 years) [2]. Pediatric inpatients accrued over $1 billion in total charges for TBI-associated hospitalizations [2]. Since TBI can have a more devastating outcome on a child’s brain, as compared with the same injury on an adult’s brain , the full effect of a traumatic injury on a child’s brain only becomes apparent over time as the brain fails to mature in step with the child’s physical growth and development [3,4,5]. Even mild TBI with imaging finding also has some serious consequences, like high incidence of temporal lobe injury (particularly in the medial temporal region) which lead to memory disorders [6].

TBIs in children are a serious problem threatening pediatric health and exacerbating the social and economic burden [7]. A continuing and pressing need exists to develop a TBI surveillance database to evaluate the effectiveness of existing treatments and to track trends in TBIs [8]. We searched ClinicalTrials.gov [9], a registry and results database of publicly and privately supported clinical studies conducted around the world on human participants, and found more than 310 pediatric-TBI clinical trials that were conducted in the U.S. from 2000 to 2016 with an additional 10 clinical trials recruiting pediatric patients with TBI or a concussion from U.S. hospitals emergency departments (EDs). Recruitment of sufficient numbers of children with TBI as study participants is an ongoing challenge facing researchers and clinicians who plan clinical trials [8]. In addition, in its 2014 Special Report to Congress, the Centers for Disease Control and Prevention (CDC) identified major gaps in TBI surveillance [10]. These gaps include: insufficient sample size of regional or state TBI surveillance, a lack of uniform race and ethnicity data in TBI data systems, and TBI data not being readily available in states not funded by the CDC. To address these gaps, the CDC recommended a new strategy to investigate TBI-related medical encounters by using data from the Healthcare Cost and Utilization Project (HCUP). 

This study’s main objective is to provide national estimates of ED medical encounters for pediatric TBIs in the U.S. and to describe patient and hospital characteristics. Due to a high false positive diagnosis using the unspecified head injury International Classification of Disease, Ninth Revision, and Clinical Modification (ICD-9-CM 959.01), we investigated the effect of the ICD-9-CM 959.01 on the national TBI estimates and age/gender patterns of pediatric TBIs. We also estimated sample sizes available for future clinical trials of pediatric TBI in U.S. hospital EDs.

## 2. Materials and Methods

### 2.1. Data Sources

The 2006–2013 Nationwide Emergency Department Sample (NEDS) is part of the Healthcare Cost and Utilization Project (HCUP) developed by the Agency for Healthcare Research and Quality (AHRQ). The NEDS is the largest all-payer ED database that is publicly available in the U.S. Based on a stratified probability sample of hospital-based EDs from community, non-rehabilitation hospitals, the NEDS contains about 30 million ED visits from more than 900 hospitals that approximate a 20-percent stratified sample of U.S. hospital-based EDs in 2013. The NEDS provides more than 100 clinical and non-clinical variables for each ED visit, which includes up to 15 ICD-9-CM codes.

The NEDS uses a stratified and random sampling scheme. The universe of the NEDS is defined as (1) community, non-rehabilitation hospital-based EDs in the U.S. that were included in American Hospital Association Annual Survey database, and (2) reported total ED visits. After stratifying and sorting hospitals into the universe, a random sample of up to 20 percent of the total number of hospital-based EDs was selected within each stratum (defined by region, trauma designation, urban-rural location, teaching status, and hospital ownership, or control). Strata and weighting variables were provided for making national estimates of disease-specific medical ED encounters.

### 2.2. Definition of Pediatric TBI

TBI cases were identified using the ICD-9-CM diagnosis code, recommended by the CDC for TBI surveillance. All visits in the NEDS database were screened for a TBI ICD-9-CM code. The CDC recommended TBI definitions include the following ICD-9-CM diagnosis codes: 800.0–801.9 (fracture of the vault or base of the skull); 803.0–804.9 (other and unqualified and multiple fractures of the skull); 850.0–854.1 (intracranial injury including concussion, contusion, laceration, and hemorrhage); 950.1–950.3 (injury to the optic chiasm, optic pathways, and visual cortex); 959.01 (head injury, unspecified); and 995.55 (shaken infant syndrome). ED visits were defined as TBI injuries if any of the diagnosis codes met the above CDC TBI definition, regardless of whether other diagnoses were present [11].

### 2.3. Definition of Pediatric Concussion

Concussion is a subgroup of TBIs. In this study, concussion cases were defined based on ICD-9-CM diagnosis code (ICD-9-CM 850.x).

### 2.4. Definition of Pediatric Severe TBI

Severe TBI cases were defined as TBI patients with head Abbreviated Injury Scale (AIS) greater than or equal to 3 [12].

### 2.5. Definition of TBI Type

TBI type are defined as: Type 1 TBI: if there is recorded evidence of an intracranial injury or a moderate or a prolonged loss of consciousness (LOC), Shaken Infant Syndrome (SIS), or injuries to the optic nerve pathways; Type 2 TBI: includes injuries with no recorded evidence of intracranial injury, and LOC of less than one hour, or LOC of unknown duration, or unspecified level of consciousness; Type 3 TBI: includes patients with no evidence of intracranial injury and no LOC, ISS, Injury Severity Score.

### 2.6. Study Variables

Study variables extracted from NEDS included 15 ICD-9-CM diagnosis, Injury Severity Score, age, sex, median household income, primary expected payer, patient location, major external cause (defined by first E-code), trauma center level designation, hospital urban-rural designation, control/ownership of hospital, region of hospital, and teaching status of hospital. 

We used the ICD Programs for Injury Categorization (ICDPIC) (Version 3.0 requiring STATA 14.0 software, College Station, TX, USA) to generate AIS for each TBI case from 2006 to 2013. 

### 2.7. Statistical Analysis

Data analyses were conducted using the SAS^®^ Studio 3.4 Mid-Tier (the Enterprise edition) (SAS Institute Inc., Cary, NC, USA). All data were weighted and then adjusted to represent all pediatric visits in community, non-rehabilitation hospital-based EDs in the U.S. Sample size and weighted national estimates with 95% confidence intervals (CIs) were calculated for pediatric TBI, concussion, and severe TBI from 2006 to 2013. 

Patient demographics and hospital characteristics in 2013 were analyzed using weighted percentages with 95% CIs. We then calculated the rate of pediatric TBI-related ED visits per 1000 U.S. children using age and gender-specific population data as the denominator. Age and gender population data were from the 2013 U.S. Census Bureau [13]. 

A separate analysis also produced a national estimate of pediatric TBI-related ED visits by excluding unspecified head injury cases (ICD-9-CM code 959.01) to show the effect of unspecified head injuries on our findings.

### 2.8. Bootstrap Simulation Analysis

To estimate the annual number of pediatric TBI, concussion, and severe TBI cases for patient recruitment from randomly selected community, non-rehabilitation hospital-based EDs for future clinical trials, we performed bootstrap simulation analyses. First, we randomly selected 5 and 10 hospitals and sampled without replacement for a total of 1000 replicates from all hospitals with annual TBI-related ED visits greater than 500. Next, we calculated median, 5th percentile, and 95th percentile of children with TBI, concussion, and severe TBI diagnoses over these 1000 replicates. Similar bootstrap simulation analyses were repeated for hospitals with the following characteristics: annual TBI-related ED visits >1000, Level I Trauma Center, Level II Trauma Center, pediatric hospitals, adult hospitals, teaching hospitals, non-teaching hospitals, and by U.S. region (Northeast, Midwest, Southern, and Western).

## 3. Results

Between 2006 and 2013, of the estimated 215,204,932 pediatric ED visits in the U.S. from the NEDS database, 6,089,930 (2.83%) had a diagnosis of pediatric TBI when the ICD-9-CM diagnosis code for unspecified head injury (code 959.01) was included. ED visits for pediatric TBI (including 959.01 diagnoses) increased by 34.1% from 624,705 visits in 2006 to 837,428 visits in 2013 after a maximum increase of 44.9% (905,225 visits) in 2012 (Table 1). The observed trends for subgroups excluding the unspecified head injury diagnosis (code 959.01) and for pediatric concussion, also increased by 21.6% and 35.2% respectively from 2006 to 2013, again after maximums of 25.9% and 41%, respectively in 2012 (Table 1). Conversely, the trend for medical encounters for severe pediatric TBI saw a decline by 18.4% from 2006 to 2013 with the greatest decline (18.9%) observed in 2012 (Table 1).

Patient demographics and hospital characteristics of pediatric TBI subgroups (including and excluding code 959.01 diagnoses, concussion, and severe TBI) were extracted from NEDS for 2013 (Table 2). Of all subgroups with pediatric TBI, children <1 year (20.9%) had a significantly higher percentage of severe TBI (Table 2). The differences in hospital characteristics among pediatric TBI, concussion, and severe TBI diagnoses—with the exception of pediatric hospitals and hospital region—were statistically insignificant. Pediatric hospital EDs treated a higher proportion of severe pediatric TBI (19.8%), as compared with diagnoses of pediatric TBI or concussion, which each averaged about 9% of their TBI caseloads (Table 2). Hospitals located in the Northeast treated a significantly lower proportion of pediatric severe TBI cases (10.4%), as compared with diagnoses of pediatric TBI or concussion, which each averaged about 20% of their TBI caseloads (Table 2). 

**Figure 1:**
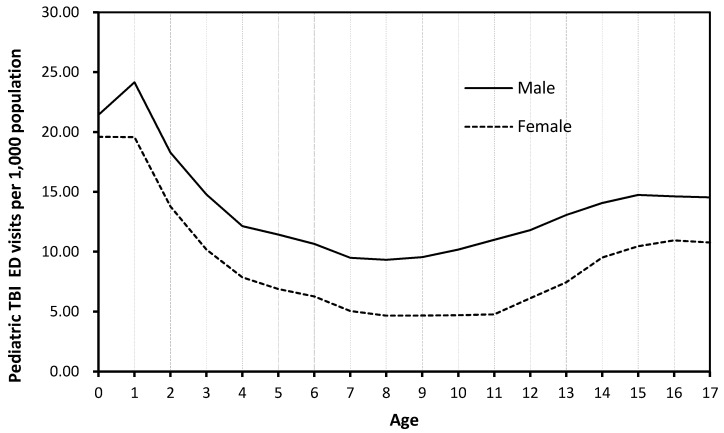
Rate of pediatric TBI visits (ICD-9-CM 959.01 included) by age and gender, 2013 NEDS.

**Figure 2:**
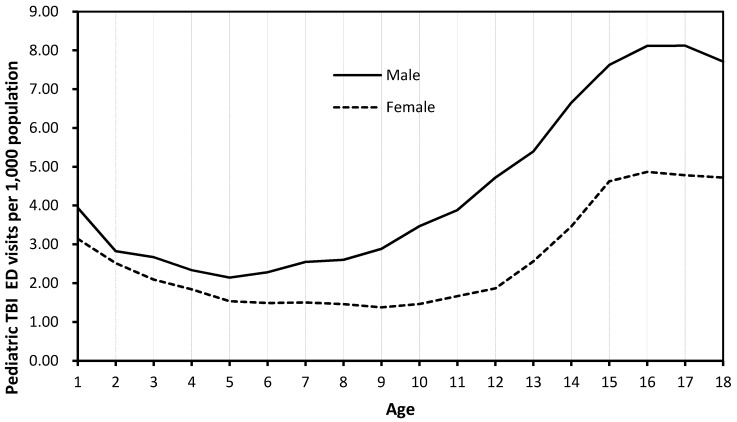
Rate of pediatric TBI visits (ICD-9-CM 959.01 excluded) by age and gender, 2013 NEDS.

Rates of pediatric TBI visits by age for both genders differed significantly depending on if unspecified head injury cases (ICD-CM code 959.01) were included (Figure 1) or excluded (Figure 2). When unspecified head injury cases (code 959.01) were included, infants and toddlers (less than 2 years) had the highest rate of TBI diagnoses, while children (8–10 years) had the lowest TBI rate (Figure 1). However, when unspecified head injury cases (code 959.01) were excluded, adolescents (15–18 years) had the highest TBI incidence rate and children (5–8 years) had the lowest rate (Figure 2). 

To facilitate researchers in estimating sample sizes for future clinical trials, bootstrap simulation analyses were conducted to estimate potentially available pediatric TBI, concussion, and severe TBI cases from hospital EDs of different characteristics (Table 3). Our results provided the potential number of TBI cases available for clinical trials under different circumstances. The number of estimated severe pediatric TBI cases were significantly fewer than TBI (excluding code 959.01 diagnoses) or concussion cases, suggesting that clinical trials targeting severe pediatric TBI will likely face a challenge in recruiting sufficient severe TBI patients into their trials.

## 4. Discussion

Between 2006 and 2013, the rate of increase in pediatric TBI-related ED visits in the U.S. was eight-fold greater than the rate of increase of total pediatric ED visits (Table 1). The Centers for Disease Control and Prevention (CDC) analyzed the National Hospital Ambulatory Medical Care Survey data [14] and also reported TBI-related ED visits increased for all age groups from 2001 to 2010. Young children (0 to 4 years) have the highest rates of any age group, which are almost twice the rate of teens to young adults (15–24 years) [14]. The rising trend of pediatric TBI-related ED visits in the U.S. is disturbingly apparent. This increase of pediatric TBI ED visits was largely due to increased visits for concussion and unspecified head injuries (Table 1) [15]. The reasons for this observed increase are unknown, but several factors may have contributed. First, there has been a growing awareness among parents, players, coaches, athletic trainers, and the general public about the potentially serious consequences of concussion [16]. Second, many states have passed youth sports concussion laws since 2009 [16]. Third, it could be possible that the introduction of the electronic medical record improved access to ICD-9 codes for concussion and other TBIs [17]. By contrast, severe pediatric TBI-related ED visits exhibited a clear decreasing trend from 2006 to 2013 (Table 1). For clinical trials that need to recruit participants, bi-directional changes in pediatric TBI ED visits suggest that recruiting children with concussion will likely get easier, whereas recruiting children with severe TBI will get harder.

A recognized challenge in accurately identifying TBI cases is the use of the ‘unspecified injury to the head’ diagnostic code (code 959.01). The International Classification of Diseases (ICD) is considered the “standard diagnostic tool for epidemiology, health management, and clinical purposes” [18], and specific ICD9-CM codes are recommended by the CDC to identify TBI cases in TBI surveillance based on medical records data. Bazarian and colleagues (2006) examined the ICD-9-CM case definition for TBI and found that the ‘unspecified’ ICD-9-CM code 959.01 represented 58% of all TBI cases; 62.4% of these ‘unspecified’ cases were false positives [19]. In our study, most of the children diagnosed with ‘unspecified injury to the head’ were infants (aged zero to one year), which explained the overall patterns of rate of pediatric TBI-related ED visits by age (Figure 1). Infants and toddlers (aged less than two years) had the highest TBI incidence rate. Excluding the unspecified head injury code 959.01 cases would substantially change the epidemiological patterns of pediatric TBIs in the U.S. (Figure 2). Chan et al. (2015) showed that inclusion of “unspecified injury to the head” diagnostic code 959.01 data could result in significant changes in estimated TBI cases, healthcare utilizations, and causes of TBI [20]. Therefore, careful assessment of inclusion of “unspecified injury to the head” diagnostic code 959.01 in the TBI case definition in future TBI surveillance and research is critical. Inclusion vs. exclusion of code 959.01 diagnoses will have implications for estimating TBI cases, resource allocation, healthcare services planning, and intervention priority setting [20].

Investigators planning for clinical trials of pediatric TBI need an improved methodology for projecting the required number of study sites and potential patient cases. In this study, we described the number and distribution of pediatric TBI, concussion, and severe TBI ED visits from a representative sample of EDs in the U.S. Our research yielded an important observation for future pediatric TBI clinical trials. When unspecified head injuries (code 959.01) are excluded, the majority of pediatric TBI-related ED visits occurred at hospital EDs that had annual TBI ED visits >1000, and were levels I and II trauma centers, pediatric hospitals, or teaching hospitals. Likewise, pediatric concussion cases were more likely to be recruited at hospital EDs that have annual TBI ED visits >1000, and are levels I and II trauma centers, or pediatric hospitals. In contrast, very few EDs had high volumes of children with severe TBI. The estimated median of annual severe TBI-related visits in most hospital EDs was less than 50. Therefore, clinicians will have a major challenge in recruiting sufficient children with severe TBI to power their clinical trials from U.S. EDs, if only a few study sites participate.

Based on findings in our study from U.S. hospital EDs, to be maximally efficient, recruiters for future clinical trials of children with TBI should target hospitals with the following characteristics: (1) hospitals with greater than 1000 annual TBI-related ED visits, (2) level I or II trauma centers, (3) pediatric hospitals, or (4) teaching hospitals. Clinical trials recruiting children with severe TBI should focus on multiple pediatric hospitals with level I trauma center status. 

The above recommendations are purely logistical from statistical perspectives, but other practical considerations are essential when designing clinical trials. First, our data provided median, 5th percentile, and 95th percentile of the estimated numbers of pediatric TBI-related ED visits. Considerable variations in these estimates (Table 3) suggest that the actual number of children with TBI treated in U.S. hospital EDs varied significantly. Researchers should first conduct study feasibility assessments to estimate potentially available children with TBI for the proposed clinical trial. Second, recruiting children with TBI into clinical trials is known to be challenging [21]. Parents usually worry about the adverse effects of clinical trials, therefore the actual number of children with TBI whose parents agree to enroll their child in a clinical trial will be likely be fewer than projected [21]. Thus, our simulation results might have overestimated the numbers of pediatric TBI patients available for future clinical trials since the data on parental-consent-rates did not exist. Finally, because inclusion and exclusion criteria in each clinical trial are often project-specific [22], the percentage of pediatric TBI patients meeting study inclusion criteria may vary greatly in clinical trials., Future investigators need to first estimate sample size and study power of their planned clinical trials using the inclusion and exclusion criteria in the analysis of existing medical records of the participating sites.

The large number of ED records in the NEDS and the ability to generate national estimates are major strengths of this study. Our research, however, has several limitations. First, due to the nature of NEDS, we used only a single measure, head Abbreviate Injury Score [23], while others have used physiologic criteria as well as resources and interventions required during trauma patient care to identify severe injuries [24]. However, head injury AIS directly correlates with trauma patient mortality and is a common criterion for identifying severe head injury by previous researchers. Second, individual patients who visited hospital EDs multiple times in one year might have multiple records in the NEDS datasets. The NEDS has no unique patient identifier that would allow us to identify and exclude multiple ED visits. Third, because children with more severe injuries are more likely to be hospitalized and most mild TBI and concussions are treated in primary care, the rate of pediatric TBI-related ED visits reflects only partial TBI incidence in the US.

## 5. Conclusions

Our study of nationally representative ED visits data from the 2006–2013 Nationwide Emergency Department Sample (NEDS) found pediatric TBI related ED visits in the US increased by about 30% over the past decade. This increase was likely due to an increase of pediatric concussions treated in hospital EDs. Conversely, the number of severe pediatric TBI ED visits decreased during the same period. Bi-directional changes in pediatric TBI ED visits suggest that recruiting children with concussions will likely get easier, whereas recruiting children with severe TBI will get harder. Including unspecified head injury cases with ICD-9-CM code 959.01 in TBI surveillance would significantly change the national estimates and demographic patterns of pediatric TBI patients. Future clinical trials of pediatric TBI patients should first conduct a careful feasibility assessment to estimate sample size and study power using existing medical records of selected study sites.

## Figures and Tables

**Table 1:** Trend of pediatric traumatic brain injury (TBI)-related ED visits (National Estimates), NEDS ^1^ 2006–2013.

Year	Pediatric ED Visits		Pediatric TBI (Including Code 959.01) ^2^		Pediatric TBI (Excluding Code 959.01)		Pediatric Concussion		Pediatric Severe TBI	
	Weighted Number	% Change ^3^	Weighted Number	% Change	Weighted Number	% Change	Weighted Number	% Change	Weighted Number	% Change
**2006**	26,421,966	0.0	624,705	0.0	216,097	0.0	162,013	0.0	34,046	0.0
**2007**	26,952,590	2.0	638,088	2.1	224,657	4.0	168,717	4.1	35,311	3.7
**2008**	26,422,740	0.0	656,897	5.2	231,665	7.2	177,039	9.3	35,324	3.8
**2009**	28,411,936	7.5	798,044	27.7	243,015	12.5	193,538	19.5	31,893	−6.3
**2010**	25,511,069	−3.4	778,138	24.6	252,566	16.9	202,681	25.1	31,777	−6.7
**2011**	27,010,706	2.2	851,405	36.3	255,072	18.0	209,917	29.6	28,575	−16.1
**2012**	27,877,600	5.5	905,225	44.9	272,004	25.9	228,424	41.0	27,620	−18.9
**2013**	26,596,325	0.7	837,428	34.1	262,826	21.6	218,984	35.2	27,787	−18.4
**Total**	**215,204,932**		**6,089,930**		**1,957,902**		**1,561,313**		252,333	

^1^ NEDS, Nationwide Emergency Department Sample; ^2^ ICD-9-CM code 959.01 defines unspecified head injury; ^3^ Percentage change based on 2006 reference year.

**Table 2:** Patient demographics and hospital characteristics of pediatric traumatic brain injury (TBI)-related ED visits, NEDS ^1^ 2013.

		Pediatric TBI Including 959.01 ^2^ (n = 837,428)	Pediatric TBI Excluding 959.01 ^2^ (n = 262,826)	Pediatric Concussion (n = 218,984)	Pediatric Severe TBI (n = 12,370)
**Gender, weighted % (95% CI) ^3^**					
	Male	61.2 (60.9–61.5)	64.09 (63.6–64.6)	64.0 (63.4–64.5)	66.2 (64.4–68.1)
	Female	38.9 (38.6–39.1)	35.9 (35.4–36.4)	36.0 (35.5–36.6)	33.8 (31.9–35.6)
**Age (years), weighted % (95% CI)**					
	Age 0–	9.7 (9.2–10.1)	5.3 (4.6–6.1)	1.5 (1.3–1.7)	20.9 (18.3–23.4)
	Age 1–4	29.0 (28.3–29.6)	13.7 (12–14.7)	11.8 (10.9–12.7)	19.7 (18.0–21.4)
	Age 5–9	19.2 (18.9–19.5)	16.5 (16–17.1)	16.6 (16.0–17.2)	15.3 (13.8–16.9)
	Age 10–14	23.0 (22.5–23.6)	33.7 (32–34.6)	36.9 (35.9–37.8)	19.5 (17.5–21.6)
	Age 15–17	19.1 (18.4–19.9)	30.8 (29–32.3)	33.2 (32.0–34.4)	24.5 (20.3–28.8)
**TBI Type, weighted % (95% CI)**					
	Type 1	9.9 (8.5–11.4)	9.9 (8.5–11.4)	0.2 (0.1–0.2)	88.0 (86.5–89.5)
	Type 2	85.6 (83.8–87.5)	85.6 (83.8–87.5)	99.8 (99.8–99.9)	4.6 (3.7–5.5)
	Type 3	4.4 (3.8–5.0)	4.4 (3.8–5.0)	-	7.4 (6.3–8.5)
**ISS, weighted % (95% CI)**					
	ISS 1–3	3.1 (2.9–3.3)	9.9 (9.3–10.6)	11.0 (10.2–11.7)	-
	ISS 4–8	92.5 (91.8–93.1)	77.5 (75.8–79.1)	87.1 (86.3–87.9)	-
	ISS 9–15	2.9 (2.6–3.3)	7.9 (6.9–8.9)	1.6 (1.5–1.8)	-
	ISS 16–24	1.3 (1.1–1.4)	3.9 (3.4–4.4)	0.3 (0.2–0.4)	84.3 (82.3–86.3)
	ISS 25–75	0.2 (0.2–0.3)	0.7 (0.6–0.9)	0.1 (0.0–0.1)	15.7 (13.7–17.7)
**Major external cause, weighted % (95% CI)**					
	Fall	47.8 (46.2–49.4)	38.3 (36.7–40.0)	37.0 (35.5–38.5)	30.9 (28.4–33.4)
	Struck	30.0 (29.0–31.1)	34.1 (32.6–35.6)	38.1 (36.7–39.5)	10.0 (8.7–11.4)
	Motor vehicle crash	6.6 (6.2–6.9)	8.4 (7.8–9.0)	7.3 (6.8–7.8)	26.7 (23.7–29.8)
**Patient location, weighted % (95% CI)**					
	Large central metropolitan	27.0 (22.7–31.2)	22.7 (18.0–27.5)	21.7 (17.0–26.3)	23.7 (18.2–29.3)
	Large fringe metropolitan	25.7 (22.1–29.3)	26.0 (22.3–29.7)	26.9 (23.1–30.8)	20.9 (15.7–26.2)
	Medium metropolitan	22.1 (18.9–25.4)	21.6 (18.3–24.9)	21.6 (18.4–24.9)	21.6 (16.7–26.5)
	Small metropolitan	8.4 (6.7–10.2)	9.0 (7.2–10.8)	8.9 (7.0–10.9)	10.1 (7.8–12.4)
	Micropolitan	11.0 (9.8–12.1)	12.4 (11.2–13.7)	12.5 (11.1–13.8)	14.7 (12.2–17.2)
	Not metropolitan or micropolitan	5.8 (5.2–6.5)	8.2 (7.3–9.2)	8.3 (7.3–9.4)	8.9 (7.2–10.7)
**Primary expected payer, weighted % (95% CI)**					
	Medicare	0.4 (0.1–0.6)	0.4 (0.1–0.6)	0.3 (0.1–0.5)	0.3 (0.0–0.5)
	Medicaid	43.1 (41.4–44.9)	35.9 (34.1–37.7)	33.7 (32.1–35.3)	46.3 (42.2–50.3)
	Private including HMO	46.3 (44.5–48.2)	54.0 (52.0–55.9)	56.1 (54.4–58.1)	43.0 (39.0–47.1)
	Self-pay	5.7 (5.4–6.1)	5.2 (4.8–5.6)	5.3 (4.8–5.7)	4.2 (3.4–5.0)
	No charge	0.0 (0.0–0.1)	0.1 (0.0–0.1)	0.1 (0.0–0.1)	0.1 (0.0–0.2)
	Other	4.4 (3.6–5.2)	4.5 (3.8–5.2)	4.3 (3.7–5.0)	6.2 (3.5–8.8)
**Children’s hospital, weighted % (95% CI)** ^4^					
	No	92.3 (86.2–98.4)	89.1 (80.6–97.6)	90.9 (83.3–98.5)	80.2 (65.9–94.4)
	Yes	7.7 (1.6–13.8)	10.9 (2.4–19.4)	9.1 (1.5–16.7)	19.8 (5.6–34.1)
**Trauma center level designation, weighted % (95% CI)**					
	Non-trauma center	37.8 (34.1–41.5)	36.6 (32.2–41.0)	39.5 (35.1–43.9)	16.4 (12.7–20.2)
	Trauma center level I	20.6 (15.0–26.2)	24.9 (17.7–32.1)	20.2 (13.5–26.9)	53.8 (45.0–62.6)
	Trauma center level II	10.9 (8.8–13.0)	10.3 (8.2–12.4)	10.4 (8.4–12.4)	11.9 (8.2–15.7)
	Trauma center level III	8.4 (6.8–9.9)	8.7 (6.9–10.5)	9.2 (7.3–11.2)	5.2 (3.8–6.6)
	Non-trauma or trauma center level III, collapsed category	17.1 (14.9–19.4)	14.7 (12.2–17.3)	16.0 (13.2–18.7)	7.2 (5.7–8.8)
	Trauma center level I or II, collapsed category	5.2 (3.6–6.9)	4.8 (3.4–6.1)	4.8 (3.5–6.0)	5.5 (3.8–7.1)
**Hospital urban-rural designation, weighted % (95% CI)**					
	Large metropolitan areas with at least 1 million residents	49.6 (45.3–54.0)	46.4 (40.8–52.0)	45.1 (39.8–50.5)	51.4 (42.1–60.8)
	Small metropolitan areas with less than 1 million residents	29.7 (26.1–33.3)	30.9 (26.4–35.4)	30.5 (26.6–34.4)	35.4 (26.0–44.7)
	Micropolitan areas	10.1 (8.7–11.4)	10.4 (8.8–11.9)	11.2 (9.6–12.9)	5.4 (4.1–6.6)
	Not metropolitan or micropolitan	4.6 (3.9–5.3)	6.6 (5.6–7.7)	7.3 (6.1–8.4)	2.4 (1.9–2.9)
	Collapsed category of small metropolitan and micropolitan	2.5 (1.5–3.5)	2.2 (1.5–3.0)	2.2 (1.4–3.0)	2.7 (1.5–4.0)
	Metropolitan, collapsed category of large and small metropolitan	2.8 (1.2–4.3)	2.4 (1.1–3.7)	2.5 (1.1–3.9)	1.4 (0.6–2.2)
	Non-metropolitan, collapsed category of micropolitan and rural	0.8 (0.5–1.1)	1.1 (0.8–1.5)	1.2 (0.8–1.6)	1.3 (0.7–1.8)
**Control/ownership of hospital, weighted % (95% CI)**					
	Government or private, collapsed category	68.9 (65.8–72.0)	69.3 (65.5–73.1)	67.0 (63.2–71.0)	84.2 (81.1–87.4)
	Government, nonfederal, public	5.5 (4.5–6.6)	5.9 (4.7–7.1)	6.4 (5.1–7.7)	2.9 (2.2–3.7)
	Private, non-profit, voluntary	16.0 (13.8–18.3)	14.9 (12.2–17.6)	16.0 (13.1–18.9)	7.6 (6.0–9.3)
	Private, invest-own	6.6 (5.5–7.7)	5.7 (4.6–6.7)	6.1 (5.0–7.2)	3.3 (2.4–4.1)
	Private, collapsed category	3.0 (2.5–3.5)	4.2 (3.4–5.0)	4.6 (3.7–5.4)	1.9 (1.3–2.5)
**Region of hospital in US, weighted % (95% CI)**					
	Northeast	20.6 (17.5–23.8)	19.4 (16.0–22.7)	20.8 (17.3–24.4)	10.4 (6.8–13.9)
	Midwest	22.9 (18.8–27.0)	25.3 (19.8–30.7)	25.7 (20.3–31.1)	23.2 (15.6–30.8)
	South	33.6 (29.7–37.5)	31.9 (27.5–36.2)	31.4 (27.3–35.4)	36.8 (28.3–45.2)
	West	22.9 (19.0–26.7)	23.5 (18.2–28.9)	22.1 (17.9–26.3)	29.6 (19.3–39.9)
**Teaching status of hospital, weighted % (95% CI)**					
	Metropolitan non-teaching	39.8 (36.0–43.5)	36.2 (31.8–40.5)	38.5 (34.2–42.8)	21.2 (16.9–25.5)
	Metropolitan teaching	44.8 (40.2–49.4)	45.7 (40.0–51.5)	41.8 (36.3–47.4)	69.7 (63.8–75.6)
	Non-metropolitan	15.5 (13.7–17.2)	18.1 (15.8–20.4)	19.7 (17.3–22.0)	9.1 (7.1–11.0)

^1^ NEDS, Nationwide Emergency Department Sample; ^2^ ICD-9-CM code 959.01 defines unspecified head injury; ^3^ CI, Confidence Interval ^4^ Children’s hospital: If the median age of all patients treated <10 then the hospital is identified as children’s hospital.

**Table 3:** Estimated numbers of pediatric traumatic brain injury (TBI)-related ED visits based on bootstrap simulation analysis.

Hospital Classification	No. of Hospitals in NEDS 2013	No. of Hospitals Selected	Number of Pediatric TBIs (Excluding 959.01) ^1^				Number of Pediatric Concussions				Number of Severe Pediatric TBIs			
			Median	P_5_–P_95_^2^			Median	P_5_–P_95_^2^			Median	P_5_–P_95_^2^		
TBI ED visits > 500	408	5	457	(202	–	1435)	396	(172	–	976)	37	(11	–	291)
TBI ED visits > 500	408	10	960	(526	–	2429)	813	(465	–	1769)	84	(33	–	396)
TBI ED visits > 1000	211	5	694	(355	–	2104)	571	(293	–	1503)	67	(22	–	330)
TBI ED visits > 1000	211	10	1429	(847	–	3180)	1183	(719	–	2651)	164	(63	–	504)
Trauma center I	49	5	1,184	(389	–	3115)	774	(259	–	2334)	251	(67	–	651)
Trauma center I	49	10	2493	(1171	–	5190)	1613	(790	–	3778)	522	(209	–	1055)
Trauma center II	46	5	657	(394	–	1181)	556	(329	–	979)	71	(29	–	161)
Trauma center II	46	10	1352	(889	–	2067)	1144	(753	–	1717)	147	(79	–	271)
Pediatric hospital	6	5	4496	(2496	–	6876)	3165	(1691	–	5376)	861	(442	–	1270)
Adult hospital	928	5	236	(70	–	645)	208	(59	–	545)	17	(3	–	102)
Adult hospital	928	10	507	(221	–	1040)	431	(190	–	870)	39	(12	–	179)
Teaching hospital	184	5	527	(177	–	2108)	424	(149	–	1700)	59	(13	–	354)
Teaching hospital	184	10	1151	(510	–	2961)	904	(414	–	2335)	153	(39	–	538)
Non-teaching hospital	363	5	265	(18	–	1244)	215	(14	–	836)	18	(5	–	60)
Non-teaching hospital	363	10	544	(82	–	2011)	425	(64	–	1440)	37	(17	–	105)
Northeast region	121	5	378	(142	–	880)	339	(129	–	765)	17	(4	–	98)
Northeast region	121	10	788	(395	–	1439)	705	(352	–	1299)	37	(14	–	138)
Midwest region	278	5	186	(56	–	649)	159	(50	–	497)	12	(2	–	117)
Midwest region	278	10	404	(163	–	2087)	348	(145	–	1763)	30	(8	–	204)
Southern region	358	5	220	(62	–	837)	189	(55	–	633)	18	(4	–	140)
Southern region	358	10	484	(204	–	1295)	411	(172	–	959)	42	(12	–	294)
Western region	183	5	278	(89	–	1348)	243	(80	–	868)	23	(5	–	306)
Western region	183	10	590	(250	–	1759)	508	(223	–	1174)	52	(16	–	357)

^1^ ICD-9-CM code 959.01 defines unspecified head injury; ^2^ P_5_, 5th percentile; P_95_, 95th percentile.
